# State Medical Society

**Published:** 1856-05

**Authors:** 


### STATE MEDICAL SOCIETY.

The following abstract of the proceedings of the State Medical Society, at its late meeting in the City of Macon, has been furnished by one of the members who attended from Atlanta.

"The annual meeting of this association took place on Wednesday, the 9th of April.

"Although there was not a very full attendance of the members, yet the meeting was one of interest and pleasure.

"The society was temporarily organized by calling Dr. L. A. Dugas to the chair, when the balloting for officers resulted in the election of Dr. Ira E. Dupree, of Twiggs, President; Dr. O'Keffe, Corresponding Secretary; Dr. Ellison, Recording Secretary; and Dr. Nottingham, Treasurer; after which, the annual address was delivered by Dr. W. W. Flewelle; andn some interesting essays read before the society. Dr. R. D. Arnold, who, at the annual meeting of the society in 1855, was appointed by the body to prepare an essay on the "Identity of yellow fever with remittent," read a very interesting paper, in which, from close observation for twenty years, the following conclusions have been arrived at: 1st. That yellow fever is a distinct fever, differing entirely from ordinary billious fever. 2d. That it is non-contagious, and not likely to be transported by a vessel, so as to affect, to any extent, the city to which the infection is carried, but that the agent is generated at the locations of all epidemics of yellow fever.

"The distinctive symptoms and post mortem appearances were referred to, and compared with those of bilious fever. The essay is one that will be read with interest by the profession. A very instructive paper, and one deserving commendation, was read by Dr. Cooper, of Americus, who was appointed at the last meeting of the society to prepare an essay on diet. Sound reasoning and good sense characterized the report. Important facts were brought forward, which are too often overlooked by the practitioner in the treatment of disease.

"The meeting, after a morning, afternoon, and evening session, concluded the business, and adjourned to assemble in Augusta, at the next annual period. Dr. Cooper was appointed to deliver the next annual address.

"It is to be regretted that more general interest is not felt by the profession, in the Medical Society of the State. It is true, a warm desire to have its advantages appreciated by physicians throughout the State has been manifested by many eminent in the profession; but there seems to be a want of that general feeling of interest among the members of our calling which is necessary to the beneficial operation of such an association. Might not a more lively interest be promoted by the encouragement of auxiliary societies in each county in the State?

"The present constitution of the State Society contemplates the formation of such, but fails to institute such a connexion as will insure that coöperation so desirable to carry out the objects of such associations. Could auxiliary societies be organized in every county where a sufficient number of physicians are conveniently located, and these societies have a representation in the State or mother society according to their number, many more would become interested. This seems to strike us as the best mode of insuring a sufficient attendance upon the annual meetings to make them desirable.

"Something is required to awaken more feeling in the matter, which if aroused, would redound to the honor of the profession, and the good of the community. J. G. W."

